# Comparison of Learning Outcomes Among Medical Students in Thailand to Determine the Right Time to Teach Forensic Medicine: Retrospective Study

**DOI:** 10.2196/57634

**Published:** 2025-02-10

**Authors:** Ubon Chudoung, Wilaipon Saengon, Vichan Peonim, Wisarn Worasuwannarak

**Affiliations:** 1 Department of Pathology, Faculty of Medicine Ramathibodi Hospital, Mahidol University, 270 Rama VI Road, Thung Phaya Thai, Bangkok, 10400, Thailand, 66 2201-1145

**Keywords:** multiple-choice question, MCQ, forensic medicine, preclinic, clinic, medical student

## Abstract

**Background:**

Forensic medicine requires background medical knowledge and the ability to apply it to legal cases. Medical students have different levels of medical knowledge and are therefore likely to perform differently when learning forensic medicine. However, different medical curricula in Thailand deliver forensic medicine courses at different stages of medical study; most curricula deliver these courses in the clinical years, while others offer them in the preclinical years. This raises questions about the differences in learning effectiveness.

**Objective:**

We aimed to compare the learning outcomes of medical students in curricula that either teach forensic medicine at the clinical level or teach it at the preclinical level.

**Methods:**

This was a 5-year retrospective study that compared multiple-choice question (MCQ) scores in a forensic medicine course for fifth- and third-year medical students. The fifth-year students’ program was different from that of the third-year students, but both programs were offered by Mahidol University. The students were taught forensic medicine by the same instructors, used similar content, and were evaluated via examinations of similar difficulty. Of the 1063 medical students included in this study, 782 were fifth-year clinical students, and 281 were third-year preclinical students.

**Results:**

The average scores of the fifth- and third-year medical students were 76.09% (SD 6.75%) and 62.94% (SD 8.33%), respectively. The difference was statistically significant (Kruskal-Wallis test: *P*<.001). Additionally, the average score of fifth-year medical students was significantly higher than that of third-year students in every academic year (all *P* values were <.001).

**Conclusions:**

Teaching forensic medicine during the preclinical years may be too early, and preclinical students may not understand the clinical content sufficiently. Attention should be paid to ensuring that students have the adequate clinical background before teaching subjects that require clinical applications, especially in forensic medicine.

## Introduction

Forensic medicine is a crucial field that intersects with the legal system. It involves the collection, analysis, interpretation, and presentation of evidence in legal cases [1]. Forensic medicine plays an essential role in assisting courts with making correct decisions by providing reliable and timely information. It also plays a critical role in protecting peoples’ rights by ensuring that their legal, civil, and human rights are upheld throughout the legal process [2]. Furthermore, studying forensic medicine is important for medical students in different countries, as they are equipped with the necessary knowledge and skills to accurately assess and document injuries and provide expert opinions on causes of death and other relevant medical information that may have legal implications [3-6].

This subject is included among the professional subjects that every Thai medical student must study to comply with the Criminal Procedure Code of Thailand, which requires physicians working in public hospitals to be able to perform postmortem inquests with police in cases where no forensic physician is available [7]. The Medical Council of Thailand has included forensic medicine as a mandatory subject in every doctor of medicine program.

The doctor of medicine programs in Thailand are 6**-**year programs conducted after graduating from high school. They are generally divided into 3 years at the preclinical level (first through third year) and another 3 years at the clinical level (fourth through sixth year). The teaching of each university’s curriculum differs in detail depending on various factors, such as the number of students, number of teachers, location, and service characteristics. Forensic medicine is subject to these differences.

Studying forensic medicine involves dealing with dead bodies, crime scenes, and traumatic injuries that can be emotionally and mentally stressful for some students [8]. A study from Saudi Arabia revealed that medical students have poor attitudes toward and awareness of the importance of forensic medicine [9]. Additionally, forensic medicine courses cover a wide range of topics, such as anatomy, physiology, pathology, toxicology, psychology, and jurisprudence, which can be difficult to master and integrate [10,11].

Students with different levels of medical knowledge may experience different forensic medicine course outcomes. In Thailand, most medical curricula are currently designed to teach forensic medicine to medical students at the clinical level (fifth year) [12-14]. However, some curricula have been designed to teach forensic medicine to medical students at the preclinical or early clinical level (third or fourth year) [15]. There are no clear guidelines regarding the level of students who should be taught forensic medicine.

This study aims to compare the learning outcomes of medical students in a curriculum that teaches forensic medicine at the clinical level and those of medical students in a curriculum that teaches forensic medicine at the preclinical level.

## Methods

### Study Design

This retrospective study was conducted to compare multiple-choice question (MCQ) scores of fifth- and third-year medical students from two medical curricula that teach forensic medicine. Both groups of students studied forensic medicine with the same instructors, used similar content, and were assessed via MCQ examinations with similar difficulty levels. The scores indicated the participants’ learning outcomes.

### Setting and Participants

#### Samples

Our samples included (1) medical students in a curriculum that teaches forensic medicine at the clinical level (fifth year) through the Doctor of Medicine Program at Ramathibodi Hospital, Mahidol University (782 students), and (2) medical students in a curriculum that teaches forensic medicine as the last subject at the preclinical level (third year) through the Joint Program for Producing More Doctors for Rural Areas, Mahidol University (281 students).

#### Sample Size Calculation

The sample size was designed to compare 2-sided differences in the MCQ percentage scores between third- and fifth-year medical students studying forensic medicine. The null hypothesis (H_0_) was that the MCQ percentage scores between third- and fifth-year medical students would not be significantly different. The alternative hypothesis (H_1_) was that the MCQ percentage scores between third- and fifth-year medical students would be significantly different.

We calculated the sample size according to a 5% type 1 error (α) and an 80% study power (1 – β). The significant difference (µ1 – µ2) and SD (σ) were set at 10 and 11, respectively, based on MCQ score data for medical students who studied forensic medicine from 2010 to 2014. The required sample size was 38 (19 participants in each group; Multimedia Appendix 1) [16]. However, this study included more participants than the calculated sample size.

### Intervention

#### Teaching Method

Both groups of medical students received on-site theoretical lectures before completing the MCQs. The content included basic knowledge of forensic pathology (including postmortem inquest, identification, time of death estimation, crime scene investigation, unnatural death, and sudden unexpected death), clinical forensic medicine (including patients who are wounded, child abuse, sexual assault, and forensic psychiatry), forensic evidence, forensic genetics, forensic toxicology, and medical law and ethics. Third-year medical students studied for 30 hours. Fifth-year medical students studied for 15 hours, using similar content that was more concise, and had the opportunity to visit a court for 3 hours. Neither group had the opportunity to attend crime scene investigations or autopsies (which they would attend later). This teaching method was performed regularly, and the authors did not intervene with any of the participants.

#### MCQ Examinations

For examinations, all teaching staff (4 staff members) created 5-option MCQs with a single best answer according to the topics they taught, including basic knowledge of forensic pathology (40% of questions), clinical forensic medicine (30% of questions), forensic evidence (5% of questions), forensic genetics (5% of questions), forensic toxicology (5% of questions), and medical law and ethics (15% of questions). The tests were designed to ensure that medical students are able to perform basic postmortem inquests, examine various types of forensic patients, produce accurate medicolegal reports, have basic knowledge of law and ethics, and understand the process of testifying in court. The MCQ examinations were structured via a balanced approach for cognitive function, allocating approximately 25% of the examination to knowledge, 30% to comprehension, 25% to application, and 20% to analysis level, according to the Bloom taxonomy. This distribution is maintained consistently from year to year. The examination was intended to have a moderate level of difficulty. Third-year medical students completed a 100-question examination in 2 hours, and fifth-year medical students completed an 80-question examination in 1.5 hours. Based on an analysis of the examination, most of the items had a difficulty level (p) in the range of 0.4 to 0.7 and a discriminatory power (r) in the range of 0.1 to 0.5. Internal consistency reliability (Kuder-Richardson Formula 20) was in the range of 0.6 to 0.7.

### Data Collection

In this study, the data were collected retrospectively for 5 years, from academic years 2010 through 2014.

### Statistical Analysis

For the comparison between the two groups, we used the means and SDs of the MCQ scores to test this study’s hypothesis that the learning outcome is different between third- and fifth-year students. Kruskal-Wallis and Mann-Whitney *U* tests were used for continuous variables with normal and nonnormal distributions, respectively [17]. The significance level was set at 5% (*P*<.05). The program used for data analysis was SPSS software (version 26; IBM Corp).

### Ethical Considerations

This study was approved by the Ethical Clearance Committee on Human Rights Related to Research Involving Human Subjects, Faculty of Medicine Ramathibodi Hospital, Mahidol University (MURA 2015/213). The need for informed consent was waived by the Ethical Clearance Committee on Human Rights Related to Research Involving Human Subjects, Faculty of Medicine Ramathibodi Hospital, Mahidol University. Data were collected by using an anonymous method—assigning numbers to all participants instead of names. No compensation was provided to participants.

## Results

From the collection of MCQ scores of medical students from academic years 2010 to 2014 who were taught forensic medicine, the scores of 1063 students were used in this study. The scores were divided into scores of third-year medical students (n=281) and scores of fifth-year medical students (n=728), as shown in Table 1.

**Table 1.** Number of students in each academic year (N=1063).

Students		Academic year					Total
		2010	2011	2012	2013	2014	
Third-year students, n (%)							
	Male	30 (2.8)	35 (3.3)	34 (3.2)	33 (3.1)	33 (3.1)	165 (15.5)
	Female	21 (2)	23 (2.2)	21 (2)	23 (2.2)	28 (2.6)	116 (10.9)
Fifth-year students, n (%)							
	Male	81 (7.6)	94 (8.8)	87 (8.2)	94 (8.8)	101 (9.5)	457 (43)
	Female	53 (5)	64 (6)	71 (6.7)	64 (6)	73 (6.9)	325 (30.6)
Total, n (%)		185 (17.4)	216 (20.3)	213 (20)	214 (20.1)	235 (22.1)	1063 (100)

**Figure 1.**
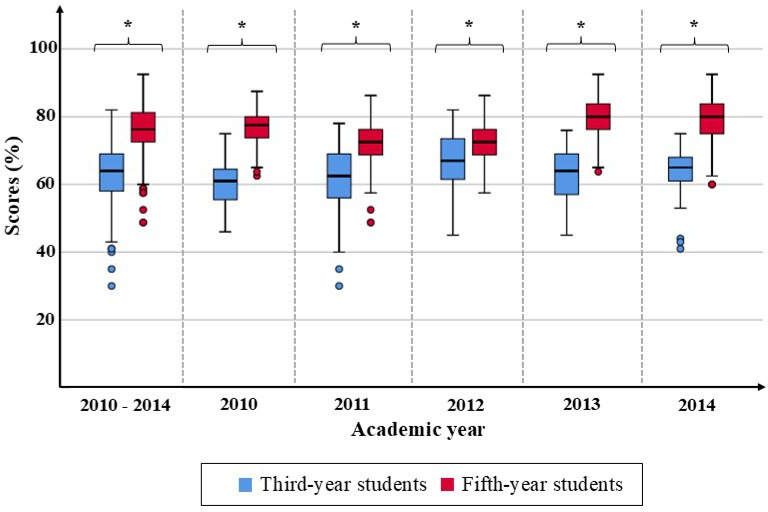
Comparing scores of third-year and fifth-year students. *Statistically significant (Mann-Whitney *U* test: *P*<.001).

When comparing students’ scores, it was found that fifth-year medical students had an average score of 76.09% (SD 6.75%), which was higher than that of third-year medical students (mean 62.94%, SD 8.33%). The difference was statistically significant (Kruskal-Wallis test: *P*<.001). In addition, when comparing the average scores in each academic year, it was found that the average score of fifth-year medical students was significantly higher than that of third-year students in every academic year (Mann-Whitney *U* test: all *P* values were <.001), as shown in Figure 1.

## Discussion

### Principal Findings

According to this study’s findings, fifth-year medical students achieved significantly higher marks on MCQs than those achieved by third-year medical students, despite the latter having more opportunities to prepare and take examinations due to their longer duration of study. The fact that the two groups of medical students had different scores may be due to their different levels of basic knowledge of medicine. Fifth-year medical students study basic clinical subjects. Therefore, they may have more comprehensive and complete basic medical knowledge and may be able to apply it to prove facts about legal cases better than third-year medical students who have not completed their basic clinical subjects. These results are consistent with a study in Italy, which showed that students’ awareness of forensic medicine improved in the fifth or sixth year of a forensic medicine course [18].

When analyzing the data by academic year, fifth-year medical students still had higher MCQ scores than those of third-year medical students, with statistical significance for each academic year. These data show that the difference in MCQ scores was unlikely due to different medical students from year to year.

In forensic medicine, students should have the opportunity to learn about real cases, including examinations of legal patients, autopsies, and crime scene examinations. This would improve students’ understanding of applying and ability to apply medical knowledge to legal applications. A study in India revealed that a court visit in a real scenario was the method that generated the most interest, and student-led objective tutorials comprised the method that best facilitated enhanced learning; the “model answer” method was also found to be an effective method for teaching forensic medicine [19]. Furthermore, a study in Mexico showed that crime scene investigation laboratory visits are an innovative method of learning that may help broaden medical students’ perspectives on forensic sciences and help them understand the multidisciplinary processes of crime investigation [20].

By integrating forensic medicine into the medical curriculum, students also gain a deeper awareness of the complexities surrounding child abuse. Training on this topic not only enhances students’ diagnostic skills but also instills a sense of responsibility to act in the best interests of the child, ensuring that they are better prepared to contribute to the early detection, intervention, and prevention of child abuse in their future careers [21].

This study used only MCQ scores from theoretical teaching, which may not measure all of the knowledge and skills of students. Although MCQs can test higher-order thinking, they are typically limited to the “application” and “analysis” levels of the Bloom taxonomy [22]. The use of MCQs is often driven by practical concerns, such as large class sizes, rather than pedagogical reasons. Although MCQs have their place, they may restrict the scope of teaching and require careful consideration to align with higher-order learning objectives [23]. Thus, a combination of test methods can be used. A study from Nepal found that objective structured practical examination is an acceptable and well-received method for medical students [24].

Integrating some content of clinical subjects via vertical integration for preclinical medical students may help to enhance their knowledge and understanding of forensic medicine. A previous study on learning environments found that undergraduate medical students from Egypt who received integrated curriculum teaching experienced a more positive learning environment [6]. Further, a similar study from Malaysia showed that integrated teaching positively affects medical students’ learning environment [25]. These studies are also consistent with guidelines from the Medical Council of Thailand for developing medical curricula in Thailand, which support horizontal and vertical integration teaching [26]; that is, clinical teachers should teach about clinical experiences from the beginning and integrate basic medical science knowledge into the clinical years.

### Limitations

A limitation of this study was its retrospective design; that is, past MCQ scores were analyzed to evaluate the medical curricula at the time of writing. No systematic interventions were conducted to test the hypothesis. In addition, this study used only MCQ scores; therefore, it may not include every learning outcome of the forensic medicine course.

### Recommendations

Students’ basic medical knowledge should be considered when teaching and learning subjects that require clinical application, especially in forensic medicine, which applies medical knowledge to law. Teaching such subjects to preclinical-level students, whose medical knowledge remains incomplete, may be too ambitious. It may be appropriate to integrate introductory content from clinical subjects to increase knowledge and understanding. In comparison, clinical-level students with complete basic knowledge may be more suitable for such clinical subjects.

### Conclusion

Forensic medicine requires basic medical knowledge and the ability to apply this knowledge in legal cases. Students’ basic medical knowledge should be considered when planning the teaching and learning of this subject. Teaching forensic medicine in the preclinical years may be too early, and doing so may result in students being unable to sufficiently understand the clinical content.

## Supplementary material

10.2196/57634 Multimedia Appendix 1 Sample size calculation.

## Abbreviations

MCQ: multiple-choice question
