# Supplementary material for: Comparison of Learning Outcomes Among Medical Students in Thailand to Determine the Right Time to Teach Forensic Medicine: Retrospective Study
Source: JMIR Med Educ. 2025 Feb 10;11:e57634. doi: 10.2196/57634 (PMC11833191; doi:10.2196/57634)
Supplement: Multimedia Appendix 1 [file mededu-v11-e57634-s001.docx]

| Multimedia Appendix 1. Sample size calculation. | |
| --- | --- |
| Reference equation [16] | Sample size calculation |
| n = (2(a+b)^2^ σ^2^) / (μ_1_-μ_2_)^2^ | n = (2(1.96+0.84)^2^11^2^) / (60-70)^2^ |
| a = conventional multiplier for alpha = 0.05  b = conventional multiplier for power = 0.80  μ_1_ = mean percentage MCQ score for   third-year medical students  μ_2_ = mean percentage MCQ score for  fifth-year medical students  μ_1_−μ_2_ = the difference the investigator wishes   to detect  σ = population variance (SD)  n = the sample size in each of the groups | a = 1.96 [Type 1 error (α) = 5%]  b = 0.84 [study power (1–β) = 80%]  μ_1_ = 60  μ_2_ = 70  μ_1_−μ_2_ = 10  σ = 11  n = 19 |
